# Low-Grade Inflammation as a Predictor of Antidepressant and Anti-Inflammatory Therapy Response in MDD Patients: A Systematic Review of the Literature in Combination With an Analysis of Experimental Data Collected in the EU-MOODINFLAME Consortium

**DOI:** 10.3389/fpsyt.2019.00458

**Published:** 2019-07-09

**Authors:** Gara Arteaga-Henríquez, Maria S. Simon, Bianka Burger, Elif Weidinger, Annemarie Wijkhuijs, Volker Arolt, Tom K. Birkenhager, Richard Musil, Norbert Müller, Hemmo A. Drexhage

**Affiliations:** ^1^Department of Psychiatry and Psychotherapy, University Hospital, Ludwig-Maximilian-University, Munich, Germany; ^2^Department of Immunology, Erasmus Medical Center, Rotterdam, Netherlands; ^3^Psychiatry, Mental Health and Addictions Group, Vall d’Hebron Research Institute (VHIR), Barcelona, Spain; ^4^Marion von Tessin Memory-Center, Munich, Germany; ^5^RMS, Rotterdam, Netherlands; ^6^Department of Psychiatry and Psychotherapy, University Hospital of Muenster, Muenster, Germany; ^7^Department of Psychiatry, Erasmus Medical Center, Rotterdam, Netherlands

**Keywords:** major depression, inflammation, antidepressant therapy, anti-inflammatory therapy, therapy prediction

## Abstract

Low-grade inflammation plays a role not only in the pathogenesis of major depressive disorder (MDD) but probably also in the poor responsiveness to regular antidepressants. There are also indications that anti-inflammatory agents improve the outcomes of antidepressants.

**Aim:** To study whether the presence of low-grade inflammation predicts the outcome of antidepressants, anti-inflammatory agents, or combinations thereof.

**Methods:** We carried out a systematic review of the literature on the prediction capability of the serum levels of inflammatory compounds and/or the inflammatory state of circulating leukocytes for the outcome of antidepressant/anti-inflammatory treatment in MDD. We compared outcomes of the review with original data (collected in two limited trials carried out in the EU project MOODINFLAME) on the prediction capability of the inflammatory state of monocytes (as measured by inflammatory gene expression) for the outcome of venlafaxine, imipramine, or sertraline treatment, the latter with and without celecoxib added.

**Results:** Collectively, the literature and original data showed that: 1) raised serum levels of pro-inflammatory compounds (in particular of CRP/IL-6) characterize an inflammatory form of MDD with poor responsiveness to predominately serotonergic agents, but a better responsiveness to antidepressant regimens with a) (add-on) noradrenergic, dopaminergic, or glutamatergic action or b) (add-on) anti-inflammatory agents such as infliximab, minocycline, or eicosapentaenoic acid, showing—next to anti-inflammatory—dopaminergic or lipid corrective action; 2) these successful anti-inflammatory (add-on) agents, when used in patients with low serum levels of CRP/IL-6, decreased response rates in comparison to placebo. Add-on aspirin, in contrast, improved responsiveness in such “non-inflammatory” patients; 3) patients with increased inflammatory gene expression in circulating leukocytes had a poor responsiveness to serotonergic/noradrenergic agents.

**Conclusions:** The presence of inflammation in patients with MDD heralds a poor outcome of first-line antidepressant therapies. Immediate step-ups to dopaminergic or glutamatergic regimens or to (add-on) anti-inflammatory agents are most likely indicated. However, at present, insufficient data exist to design protocols with reliable inflammation parameter cutoff points to guide such therapies, the more since detrimental outcomes are possible of anti-inflammatory agents in “non-inflamed” patients.

## Introduction

It is well accepted that immune dysregulation plays an important role in the pathogenesis of at least a proportion of patients with major depressive disorder (MDD) (1–16). Genetic defects and/or polymorphisms, childhood trauma, and chronic stress are all capable of eliciting such immune dysregulations (17–19). In the last decades, special interest has been raised for the role of low-grade inflammation in the immune system dysregulation of MDD. Low-grade inflammation is characterized by an increase in the level of circulating pro-inflammatory compounds, such as acute phase proteins [e.g., C-reactive protein (CRP)] and cytokines [e.g., interleukin (IL)-6], and/or by a pro-inflammatory activity of circulating or tissue resident immune cells (20–23).

A wide range of medications is currently available for the treatment of MDD. First-line agents are the well-known serotonin reuptake inhibitors (SSRIs; e.g., sertraline, escitalopram, or citalopram), which show a predominantly serotonergic action (24). First-line agents are also the serotonin-noradrenaline reuptake inhibitors (SNRIs), which show a predominantly serotonergic action at low doses and a combined serotonergic–noradrenergic action at moderate to high doses (25). Tricyclic antidepressants (TCAs) show a similar mechanism of action as SNRIs regarding the dual serotonergic-noradrenergic action, but because of more side effects, they are actually used as second-line agents. Third-line agents are drugs with a predominantly noradrenergic/dopaminergic action, such as mirtazapine or bupropion, or agents with other mechanisms of action, such as ketamine [i.e., an *N*-methyl-d-aspartate (NMDA) receptor antagonist, elevating glutamate levels]. Despite this wide range of medications, response rates to treatment are still insufficient, with about half of the patients not responding adequately to an installed treatment (26, 27).

Since most of the antidepressant drugs have—next to their neurotransmission modulatory effects—also immune modulating capacities (28, 29), it is thought that the inflammatory state of patients might play a role in non-responsiveness. To enforce the mood-regulating effects of antidepressants, and being aware of the notion that low-grade inflammation plays a role, various studies have been undertaken to use anti-inflammatory agents as add-ons to regular antidepressant therapies. In this way, acetylsalicylic acid (i.e., aspirin, a COX1 and COX2 inhibitor), selective COX-2 inhibitors (e.g., celecoxib), minocycline (a tetracyclin with anti-inflammatory effects), and anti-TNF monoclonal antibodies (e.g., infliximab) have been used experimentally (30–33). Besides these anti-inflammatory agents, agents such as cholesterol-lowering fish oil (eicosapentaenoic acid) and anti-oxidative *n*-acetylcysteine have also been used (33, 34). These agents also have anti-inflammatory actions, since both the cholesterol metabolism and the anti-oxidative machinery are linked to inflammation (35, 36). Though it seems that anti-inflammatory agents did show limited beneficial effects in most of the reported studies (30–34), there is still doubt on the real validity of such interventions, particularly due to the paucity and preliminary character of the studies, while there is also the feeling that such anti-inflammatory agents might only work in a proportion of patients.

Collectively, the abovementioned notions lead to the view that there is a need for a personalized medicine approach to select patients who, in particular, will respond to first-line agents and those needing immediate step-up therapies to drugs other than the first-line drugs and/or an add-on of a first-line agent with an anti-inflammatory agent. In such an approach, it is the question whether a pre-existent state of enhanced low-grade inflammation (present in around one-third of patients) (37) indeed plays a role in non-responsiveness to antidepressants and whether such a state is capable of predicting the outcome of the abovementioned antidepressant therapy regimens.

For this report, we have carried out a systematic review searching for the relevant literature on the prediction capability of soluble inflammatory compounds/cytokines in serum/plasma/CSF and/or the inflammatory state of circulating leukocytes for the outcome of antidepressant/anti-inflammatory treatments in MDD. We combined the outcomes of the systematic review with experimental data collected in the EU-MOODINFLAME consortium on the prediction capability of the inflammatory state of circulating monocytes (as measured by inflammatory gene expression). Two EU-MOODINFLAME trials could be evaluated, a trial carried out on patients with MDD collected at the Rotterdam site and treated in first line with venlafaxine or imipramine (38), and a small trial carried out on patients with MDD collected at the Munich site and treated with sertraline *plus* add-on celecoxib or placebo.

## Materials and Methods

### Search Strategy for Systematic Review

We conducted a systematic literature search in the PubMed/MEDLINE and Web of Science databases to identify immune-inflammatory predictors for treatment response to antidepressants, anti-inflammatory agents, and/or their combination with anti-inflammatory agents (or anti-inflammatory agents alone) in MDD from inception (for anti-inflammatory) and from 2008 (for antidepressant) until August 16, 2018. To find additional relevant studies, citation lists of included articles were tracked in Google Scholar (39) or citation lists of topic-related reviews and meta-analyses were checked. The last author of a significant paper concerning celecoxib and an expert in the field (NM) was also contacted and asked of awareness of any additional studies.

The following search terms were used: (mdd OR major depressive disorder OR depression) AND (inflammation) AND (therapy OR treatment OR antidepressant drugs OR sertraline OR venlafaxine OR escitalopram OR citalopram OR tricyclic OR ssri OR snri) AND (biomarker OR cytokines OR il-6 OR t cells OR nk cells OR th17 OR leukocytes OR macrophages OR crp OR genes) AND (response OR prediction), (mood disorder OR depression OR bipolar) AND (anti-inflammatory OR inflammation) AND (therapy OR treatment OR medication OR drugs OR add-on OR adjunct OR anti TNF OR infliximab OR CRP OR aspirin OR ASA OR acetyl salicylic acid OR minocycline OR omega 3 fatty acids OR NAC OR acetylcysteine OR cox 2 inhibitor OR celecoxib) AND (biomarker OR cytokines OR macrophages OR t cells OR NK cells OR leukocytes OR CRP OR genes).

The initial search of 7,047 studies resulted in 174 relevant studies selected by title. Inclusion criteria for further selection were:

1. publications written in the English language;
2. human clinical trials;
3. the diagnosis of MDD. Because of the paucity of studies in unipolar depression, both unipolar and bipolar depression were included for the studies on (add-on) anti-inflammatory agents. To make comparisons possible, we indicate in the result section (**Table 1C**, marked with **B** and **C**) which studies included bipolar depressed patients, and we discuss in the Discussion section putative differences stemming from this inclusion.
4. the absence of severe somatic diseases (especially inflammation-related);
5. the assessment of immune biomarkers;
6. the use of first-line or other antidepressant agents or the use of an anti-inflammatory agent added to antidepressant treatment or alone;
7. the assessment of symptom reduction with standardized measure [e.g., Hamilton Rating Scale for Depression (HAMD), Montgomery–Asberg Depression Rating Scale (MADRS), Beck’s depression inventory (BDI)] and
8. the analysis of responder and non-responder subgroups.

By reading the abstracts, methods, and results sections and applying the inclusion criteria and by removing duplicate records, 36 studies were selected. Further exclusion criteria were:

1. no predictive information provided;
2. use of parameters that are not inflammatory biomarkers in a narrower sense [e.g., serotonin and kynurenine metabolites, brain-derived neurotrophic factor (BDNF), calcium-binding protein B (S100B), macrophage-derived chemokine (MDC), platelet-derived growth factor (PDGF), and Eotaxin-1/CCL11];
3. genetic studies were excluded except for leukocyte gene expression level studies;
4. the use of agents whose anti-inflammatory mechanisms are not direct and even questionable (e.g., l-methylfolate, pioglitazone, modafinil).

By applying these exclusion criteria, we finally included 24 reports in the systematic review.

**Figure 1:**
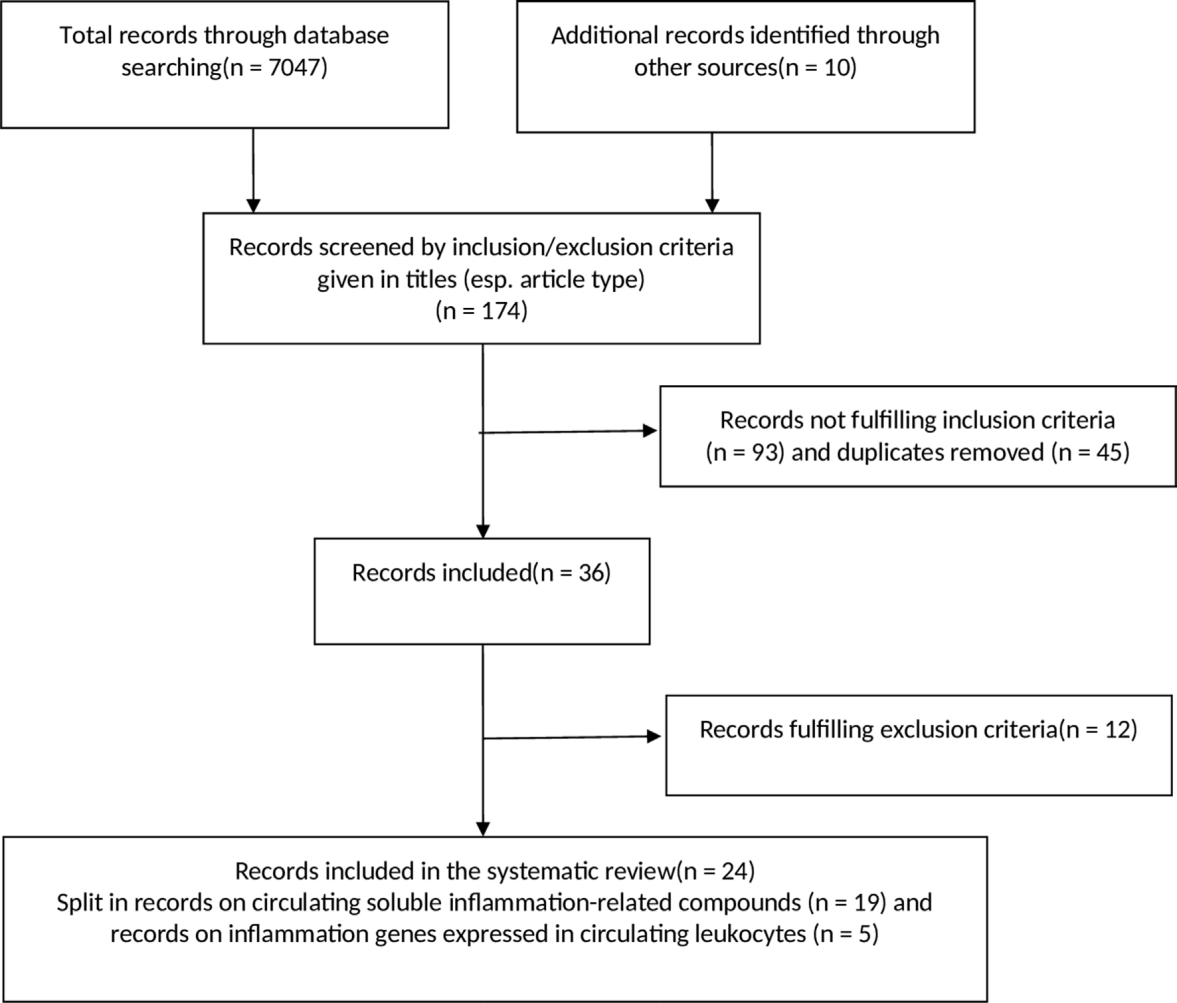
Flow diagram of the systematic research. See materials and methods section for further explanation.

With the purpose of providing a comprehensive presentation, we decided to split the remaining studies into studies concerning circulating inflammatory compounds/cytokines (*n* = 19, see **Tables 1A**–**C**) and gene expression in circulating leukocytes (*n* = 5; see **Table 2**). For detailed information about the study selection, see **Figure 1**.

**Table 1a:** Predominantly serotonergic action: higher response rates in low inflammatory state vs. moderate–high inflammatory state (prior to treatment).

INFLAMMATORY STATE	STUDY	DRUG	INFLAMMATORY TEST	RESPONSE
**LOW**	Jha et al., 2017 (40)	Escitalopram (SSRI) + Placebo	CRP < 1 mg/L	**Higher** response rates compared to m–h IS *
	Uher et al., 2014^a^ (41)	Escitalopram (SSRI)	CRP < 1 mg/L	**Higher** response rates compared to m–h IS ***
	Eller et al., 2008 (42)	Escitalopram (SSRI)	TNFα	**Higher** response rates compared to m–h IS *
**MODERATE–HIGH**	Yoshimura et al., 2013 (43)	Paroxetine, Sertraline (SSRI)	IL-6	**Higher** response rates compared to low IS *
	Manoharan et al., 2016 (44)	Fluoxetine (SSRI)	IL-6	**No associations** between biomarker values and response rates
	Jha et al., 2017 (40)	Escitalopram (SSRI) + Placebo	CRP ≥ 1 mg/L	**Lower** response rate compared to low IS *
	Chang et al., 2012 (45)	Fluoxetine (SSRI), Venlafaxine (SNRI)	CRP ≥ 1 mg/L	**Lower** response rate compared to low IS *
	Haroon et al., 2018 (46)	SSRIs, SNRIs, TCA	CRP, IL-6, TNFα, sTNF-R2	**Lower** response rate compared to low IS *
	Yoshimura et al., 2009 (47)	Paroxetine, Sertraline, Fluvoxamine, Milnacipran (SSRI, SSNRI)	IL-6	**Lower** response rate compared to low IS *
	Martinez et al., 2012 (48)	Venlafaxine (SNRI)	TNFα (CSF)	**Lower** response rate compared to low IS *

SSRI, selective serotonin reuptake inhibitors; SNRI, serotonin-norepinephrine reuptake inhibitor; TCA, tricyclic antidepressant; TNFα, tumor necrosis factor alpha; IL, interleukin; CSF, cerebrospinal fluid; CRP, C-reactive protein; m–h, moderate–high; IS, inflammatory state.

*p ≤ 0.05, **p ≤ 0.01, ***p ≤ 0.001.

a Improvement on Montgomery–Asberg Depression Rating Scale (MADRS) score 3 points higher with nortriptyline when CRP ≥ 1 mg/L and 3 points higher with escitalopram when CRP < 1 mg/L.

**Table 1b:** Predominantly noradrenergic, predominantly dopaminergic, and glutamatergic action: higher response rates in moderate–high inflammatory state vs. low inflammatory state (prior to treatment).

INFLAMMATORY STATE	STUDY	DRUG	INFLAMMATORY TEST	RESPONSE
**LOW**	Jha et al., 2017 (40)	Escitalopram (SSRI) + Bupropion (NDRI)	CRP < 1 mg/L	**Lower** response rate compared to m–h IS *
**MODERATE–HIGH**	Jha et al.,2 017 (40)	Escitalopram (SSRI) + Bupropion (NDRI)	CRP ≥ 1 mg/L	**Higher** response rates compared to low IS *
	Uher et al., 2014**^a^** (41)	Nortriptyline (TCA)	CRP ≥ 1 mg/L	**Higher** response rates compared to low IS ***
	Harley et al., 2010 (49)	Fluoxetine (SSRI) + Nortriptyline (TCA)	CRP ≥ 1 mg/L	**Higher** response rates compared to low IS ***
	Yang et al., 2015 (50)	Ketamine (NMDA Receptor Antagonist)	IL-6	**Higher** response rates compared to low IS ***
	Gupta et al., 2016 (51)	Mirtazapine (NaSSA)	TNFα	**Higher** response rates compared to low IS *

SSRI, selective serotonin reuptake inhibitors; NDRI, norepinephrine dopamine reuptake inhibitor; TCA, tricyclic antidepressant; NaSSA, noradrenergic and specific serotonergic antidepressant; TNFα, tumor necrosis factor alpha; IL, interleukin; CRP, C-reactive protein; m–h, moderate–high; IS, inflammatory state.

*p ≤ 0.05, **p ≤ 0.01, ***p ≤ 0.001.

a Improvement on Montgomery–Asberg Depression Rating Scale (MADRS) score 3 points higher with nortriptyline when CRP ≥ 1 mg/L and 3 points higher with escitalopram when CRP < 1 mg/L.

**Table 1c:** Anti-inflammatory agents (added to an antidepressant regimen, except for one study): lower response rates in low inflammatory state (prior to treatment) versus placebo and higher response rates in moderate–high inflammatory state versus low inflammatory state (prior to treatment).

INFLAMMATORY STATE	STUDY	DRUG	INFLAMMATORY TEST	RESPONSE
**LOW**	Rapaport et al., 2016 (52)	Monotherapy eicosapentaenoic acid (EPA)	e.g., IL-1ra, hs-CRP	**Lower** response rate **compared to placebo** of low inflammatory state *
	Raison et al., 2013^b^ (53)	Infliximab (anti-TNFα)	CRP ≤ 5mg/L	**Lower** response rate **compared to** placebo of low inflammatory state **
	Savitz et al., 2018^c^ (54)	Minocycline	IL-6	**Lower** response rate **compared to placebo** of low inflammatory state ^d^
	Savitz et al., 2018^c^ (54)	Aspirin (NSAID)	IL-6	**Higher** response rates compared to m–h IS ^d^
**MODERATE–HIGH**	Rapaport et al., 2016 (52)	Monotherapy eicosapentaenoic acid (EPA)	e.g., IL-1ra, hs-CRP	**Higher** response rates compared to low IS *
	Raison et al., 2013^b^ (53)	Infliximab (anti-TNFα)	CRP > 5mg/L, TNFα, sTNFR I and II	**Higher** response rates compared to low IS **
	Savitz et al., 2018^c^ (54)	Minocycline	IL-6	**Higher** response rates compared to low IS **^d^
	Husain et al., 2017 (55)	Minocycline	CRP > 5 mg/L	**Higher** response rates compared to low IS
	Porcu et al., 2018^c^ (56)	*N*-acetylcysteine	CRP > 5 mg/L	**Higher** response rates compared to low IS *
	Hasebe et al., 2017 (57)	*N*-acetylcysteine	IL-6	**No associations** between biomarker values and response rates
	Panizzutti et al., 2018^c^ (58)	*N*-acetylcysteine	CRP, IL-6, TNFα, BDNF, IL-8, IL-10	**No associations** between biomarker values and response rates
	Savitz et al., 2018^c^ (54)	Aspirin (NSAID)	CRP	**No associations** between biomarker values and response rates
	Savitz et al., 2018^c^ (54)	Aspirin (NSAID)	IL-6	**Lower** response rate compared to low IS ^d^

TNFα, tumor necrosis factor alpha; IL, interleukin; CRP, C-reactive protein; m–h, moderate–high; IS, inflammatory state.

*p ≤ 0.05, **p ≤ 0.01, ***p ≤ 0.001, reported effects without significance were not tested for significance but showed a clear descriptive trend and were therefore considered noteworthy.

^b^Mixed sample with MDD and bipolar depressed patients, ^c^Bipolar depressed sample (type I/II and unspecified), ^d^Personal communication.

**Table 2:** The predictive capability of inflammatory state prior to therapy measured by circulating leukocyte gene expression for the response to various antidepressant regimens in MDD.

ANTIDEPRESSANT AGENT	GENE TRANSCRIPT	EFFECT	STUDY
Escitalopram (SSRI)	TNF	Higher levels in non-responders	Powell et al., 2013 (59)
Escitalopram (SSRI)	13-gene model, including immune/inflammatory genes (CD3D, CD97, IFITM3, and GZMA)	Predicting non-remission with 79.4% accuracy	Guilloux et al., 2015 (60)
Escitalopram(SSRI) or Nortriptyline (TCA)	IL1β, TNF, and MIF (relative mRNA values)	Higher levels in non-responders	Cattaneo et al., 2013 (61)
Escitalopram (SSRI) or Nortriptyline (TCA)	IL1β and MIF (absolute mRNA values)	Algorithm predictive of non-response with probability of over 99%	Cattaneo et al., 2016 (62)
Antidepressant treatment, not specified	IL1β, TNF, PPT1, and HIST1H1E	Algorithm predictive of treatment response	Belzeaux et al., 2012 (63)

SSRI, selective serotonin reuptake inhibitors; TCA, tricyclic antidepressant; TNF, tumor necrosis factor; IL, interleukin; CD, cluster of differentiation; mRNA, messenger ribonucleic acid; PPT1, palmitoyl-protein thioesterase 1; HIST1H1E, histone cluster 1 H1 family member E; MIF, macrophage inhibition factor; IFITM3, interferon-inducible transmembrane protein 3; GZMA, granzyme A.

### Experimental Clinical Studies

Details on the inclusion and exclusion criteria, as well as on the clinical instruments and characteristics of patients, have been published before (38, 64). In short, in- and outpatients were recruited from the Departments of Psychiatry at the Erasmus Medical Centre (ErasmusMC) in Rotterdam (The Netherlands) and at the University Hospital of the Ludwig Maximilian University (LMU) in Munich (Germany). All patients were diagnosed according to the *Diagnostic and Statistical Manual of Mental Disorders, Text Revision* (*DSM-IV-TR*) (65) and confirmed by using the Structured Clinical Interview for DSM-IV Axis I Disorders (SCID-I) (66). Included were patients with a minimum score of 17 (Rotterdam) or 22 (Munich) on the Hamilton Rating Scale for Depression (HAMD, 17-item-version) (67).

Studies had been approved by the ethics committee of the medical faculty at the LMU, Munich (Germany), and the medical ethics committee of the ErasmusMC, Rotterdam (the Netherlands). The study was conducted in compliance with standards of Good Clinical Practice (CGP), assuring that the rights, safety, and well-being of patients were protected in accordance with the principles that have their origin in the Declaration of Helsinki (June 1964, last amendment Fortaleza 2013). Additionally, the relevant national and European regulations were adhered, too. After study procedures had been fully explained, all subjects provided written informed consent.

#### Healthy Controls

Healthy controls (HCs) were recruited from the same communities (Rotterdam and Munich). Details on the HC can be found in Refs. (64) and (68). In short, the inclusion criteria for HC were the absence of major *DSM-IV-TR* Axis I disorders including schizophrenia, psychotic disorders, mood disorders, anxiety disorders, or substance-related disorders according to *DSM-IV* criteria; the absence of usage of psychiatric drugs; and the absence of severe medical illness. HC had to be in self-proclaimed good health and free of any obvious medical illness for at least 2 weeks prior to the blood withdrawal, including acute infections and allergic reactions.

#### Treatment Protocols

Being both double-blind studies, subjects, investigators, and study staff had been blinded to the treatment assignment for the duration of the study.

##### Venlafaxine/Imipramine Study (Rotterdam)

Prior to the start of antidepressants, patients with MDD underwent a wash-out period for at least 1 week. The use of benzodiazepines was allowed up to a maximum daily dose of 3 mg lorazepam or the corresponding equivalent. Subsequently, patients were randomly assigned to a 7-week monotherapy with either the serotonin-norepinephrine reuptake inhibitor (SNRI) venlafaxine (mean daily dose 371 mg, range dose of 300–375 mg/day) or with the TCA imipramine (mean dose 206 mg, range dose of 50–450 mg/day). The duration of the treatment trial was 7 weeks to ensure that patients treated with imipramine had adequate plasma levels for at least 4 weeks. Response to treatment was defined as ≥50% reduction of the initial HAM-D score.

##### Sertraline Plus Placebo/Celecoxib Study (Munich)

Prior to the start of treatment, patients with MDD underwent a washout period for 3 days. The use of lorazepam or zopiclon was allowed in this period and also during the study, up to a maximum daily dose of 3 or 15 mg, respectively. Subsequently, patients were randomly assigned in a 1:1 ratio to a 6-week therapy with either the selective serotonin reuptake inhibitor (SSRI) sertraline *plus* placebo, or with sertraline *plus* the selective COX-2 inhibitor celecoxib. The dose of sertraline was flexible and ranged between 50 and 100 mg/day. A daily dose higher than 100 mg was not recommended, but in the expectation of more clinical benefit, a daily dose of 150 mg sertraline was allowed. The daily dose of celecoxib was 400 mg (200 mg in the morning and 200 mg in the evening). Patients from the placebo group received two identical capsules (morning and evening). As in the Rotterdam cohort, response to treatment was defined as ≥50% reduction of the initial HAM-D score.

#### Numbers of Patients With MDD and HC

Only patients and HC with full data regarding the expression levels of all key genes for monocyte inflammatory activation could be used for the present study. The Rotterdam sample therefore consisted of 34 MDD patients and 45 HC. Of the patient group, 14 patients were treated with venlafaxine and 20 patients were treated with imipramine. The Munich sample consisted therefore of 35 MDD patients and 42 HC. Of the patient group, 19 patients were treated with sertraline *plus* placebo, and 16 patients were treated with sertraline *plus* celecoxib.

#### Blood Collection

Blood was collected in sodium-heparin tubes (36 ml) for immune cell preparation just prior to treatment. From the heparinized blood, peripheral blood mononuclear cell (PBMC) suspensions were prepared by low-density gradient centrifugation *via* Ficoll-Paque PLUS (GE Healthcare, Uppsala, Sweden) within 8 h to avoid erythrophagy-related activation of the monocytes. PBMCs were frozen in 10% dimethylsulfoxide and stored in liquid nitrogen. This enabled us to test immune cells of patients and controls together at a later stage. Tests were done at ErasmusMC.

#### Monocyte Inflammatory Gene Expression

CD14^+^ monocytes were isolated from aliquots of the frozen and thawed PBMCs by a magnetic cell sorting system (auto MACS Pro, Miltenyi Biotec, B.V., Bergisch Gladbach, Germany). The average viability was 86.3 ± 10.4 (Trypan blue staining) and the purity of monocytes was 95.1 ± 3.0% (flow cytometry). RNA was isolated from the purified monocytes using RNA easy mini kit according to the manufacturer’s instructions (Qiagen, Hilden, Germany). On average, monocytes cell yield after isolation was 2.0 ± 1.6 × 10^6^/subject and the quantity of RNA in monocytes was 3.2 ± 1.8 μg. One microgram of RNA was reverse-transcribed using the cDNA high capacity reverse transcription kit (Applied Biosystems, Foster City, CA, USA). qPCR was performed using Taqman Arrays, format 48 (Applied Biosystems), according to the manufacturer’s protocol and validated against the single RT-qPCR method. Per fill port, 400 ng of cDNA (converted from total RNA) was loaded. PCR amplification was performed using an Applied Biosystems Prism 7900HT sequence detection system with TaqMan Array block. Thermal cycler conditions were 2 min at 50°C, 10 min at 94.5°C, 30 s at 97°C, and 1 min at 59.7°C for 40 cycles.

Based on several previous studies on mood disorders (21, 64, 69), we decided to include in our panel the most consistently abnormally expressed inflammatory genes in the studies. Therefore, relative to the housekeeping gene ABL1, the expression of a total of up to 49 genes was determined (also because of the maximum of fill ports in the Taqman assay) and expression values were calculated using the comparative threshold cycle (CT) method [see, for technical details, Refs. (21, 64, 69)]. The mentioned earlier studies also carried out a hierarchical clustering of these genes and found two main distinct clusters of gene expression. The first cluster is found consistently in virtually all of our monocyte inflammatory gene expression studies (also besides disease conditions such as mood disorders), and this cluster is composed of well-known pro-inflammatory cytokines and chemokines and important enzymes or transcription factors to produce these compounds. For the calculation of the “positivity of this inflammatory compound cluster”, we took the expression level of the top 10 genes [the most consistently overexpressed genes in all our studies thus far; see Ref. (64)] of this cluster into consideration, i.e., *IL1β*, *CCL20*, *EREG*, *IL6*, *TNFAIP3*, *CXCL2*, *PDE4B*, *ATF3*, *PTX3*, and *IL1A*. These genes accounted for 70–99% of the inflammatory cluster response. For each of the 10 genes, we determined a range of the HC gene expression (using the 2^−ΔCt^ values). The range was defined by the mean of the values for that gene in HC monocytes ± 1 standard deviation (SD). Then, we used this range as a standard of comparison for the MDD patients’ gene expression. We decided to refer to a patient’s top gene as upregulated, if the patient’s gene expression was higher than HC’s mean plus 1×SD, or downregulated when it was lower than HC’s mean minus 1×SD. This was done for all 10 above given genes. Then, we declared the monocyte population of a given patient as “pro-inflammatory positive” if 6 of these 10 top inflammatory genes (or more) were upregulated. These data are given in **Table 3** in the Results section. Similar calculations/algorithms for monocyte inflammatory positivity have been used by us before (21, 69–71). Further methodological details of the calculation can be found in these publications. Original Q-PCR data have been uploaded and can be retrieved *via* the GEO repository ref number GSE132315: http://www.ncbi.nlm.nih.gov/geo/query/acc.cgi?acc=GSE132315

**Table 3:** Proportions of patients with a positive inflammatory gene signature of circulating monocytes prior to therapy measured in total and by response.

**Predominantly serotonergic agents**	HC	MDD	Inflammatory negatives		Inflammatory positives	
	Positive	Positive	Responders	Non-responders	Responders	Non-responders
Sertraline *plus* Placebo (SSRI)	5/42 (12%)	7/19 (37%)	8/12 (67%)	4/12 (33%)	5/7 (71%)	2/7 (29%)
Sertraline *plus* Celecoxib (COX-2 inhibitor)	idem	3/16 (19%)	11/13 (85%)	2/13 (15%)	2/3 (67%)	1/3 (33%)
Venlafaxine (SNRI)	2/22 (9%)	6/14 (43%)	3/8 (38%)	5/8 (62%)	2/6 (33%)	4/6 (66%)
Imipramine (TCA)	2/23 (9%)	9/20 (45%)	4/11 (36%)	7/11 (64%)	2/9 (22%)	7/9 (78%)
**SUM**	9/87 (10%)	25/69 (36%)*	26/44 (59%)*	18/44 (41%)*	11/25 (44%)*	14/25 (56%)*

SSRI, selective serotonin reuptake inhibitors; SNRI, sertraline–noradrenaline reuptake inhibitors; TCA, tricyclic antidepressant; COX-2, cyclooxygenase 2; HC, healthy controls; MDD, major depressive disorder.

*p ≤ 0.05 compared to HC.

Positivity was defined by an upregulation of 6 (or more) of the 10 cluster 1 genes.

Original Q-PCR data have been uploaded and can be retrieved via the GEO repository ref number GSE132315: http://www.ncbi.nlm.nih.gov/geo/query/acc.cgi?acc=GSE132315

#### Statistics

Statistical analyses were performed using IBM SPSS v.21 for Mac. Continuous sample characteristics are reported as mean (± standard deviation). Group comparisons (e.g., MDD vs. HC, responders vs. non-responders) were analyzed using analysis of variance (ANOVA) tests for continuous data (e.g., age) and using Pearson’s chi-square (χ²) tests for categorical data (e.g., gender). For group comparisons of positivity of monocyte gene expression (e.g., MDD vs. HC, responders/non-responders vs. HC, responders vs. non-responders), Pearson’s chi-square (χ²) tests were applied, too. All hypotheses were tested with α ≤ 0.05 (two-sided).

## Results

### Systematic Review Data on the Usefulness of Circulating Serum Inflammatory Compounds

**Tables 1A**, **1B**, and **1C** show the data of the systematic review of the 19 selected articles (see the section Search Strategy for Systematic Review) regarding the predictive capability of inflammatory state [assessed by serum/plasma immune compounds (mainly CRP and cytokines) (only one study used CSF)] in patients with MDD for the response rates to various classes of antidepressant drugs and to anti-inflammatory agents added to an antidepressant regimen (except for one study, in which the anti-inflammatory agent was used as monotherapy) (52). For comprehensibility, we have grouped the outcomes in **Table 1** according to the regimen used.

**Table 1A** shows that, in three out of three studies (40–42), antidepressants with a predominant serotonergic action [i.e., escitalopram (SSRI)] induced a better response in patients with low inflammatory markers as compared to patients with high inflammatory markers in the same study. On the contrary, when inflammatory markers were high, five out of seven of the studies (40, 45–48) showed that drugs with a predominant serotonergic action (i.e., SSRIs, SNRIs, and TCAs) induced reduced response rates as compared to patients with low inflammatory markers in the same study. Cutoff points for low and high levels were defined for CRP in the reviewed studies at 1 mg/L; for IL-6 and TNFα, values depended on the actual sensitivity of the assay used in the report. Two studies formed an exception. Manoharan et al. (44) did not find any effect of pre-selection of the inflammatory state. However, this study was special in that treatment duration was of only 6 weeks, and patients had a relatively low to moderate depression severity (HAMD score ≥ 13). The other study (43) showed the opposite message (i.e., an improved response to SSRIs in patients with a high inflammatory state as compared to a low inflammatory state). This study was special, in that many patients were treated with paroxetine (SSRIs), which—apart from its serotonergic action—also exerts a considerable dopaminergic action (72).

Taken together, predominantly serotonergic agents showed, in general, insufficient response rates in those patients with signs of moderate to high inflammation as measured by circulating inflammatory compounds.

The review also delivered that, in such conditions of moderate to high signs of inflammation, drugs with another mechanism of action than primarily serotonergic do show an effect. Using nortriptyline, mirtazapine, or ketamine alone, or combinations of an SSRI with nortriptyline or bupropion resulted, in 5 out of 5 studies, in improved responses rates (40, 41, 49–51) as compared to the patients with low inflammatory markers (**Table 1B**).

Similar beneficial effects existed for combinations of antidepressant drugs with anti-inflammatory agents. **Table 1C** shows that five out of seven studies (52–56) found a significant improvement of an (add-on) anti-inflammatory therapy, when patients with high signs were compared to patients with low signs of inflammation. The anti-inflammatory agents used in these studies were infliximab, minocycline, *n*-acetylcysteine, and fish oil (the latter as monotherapy, and compared to placebo).

It must be mentioned that the study of Savitz et al. (54) only noted such improving effect with minocycline; aspirin had no such effect in their study. Aspirin did work in their “non-inflamed” patients, yet had no effect or even a reduced effect in patients with high signs of inflammation, depending on the inflammatory serum marker used to determine the state of low-grade inflammation (CRP or IL-6, see **Table 1C**). The study of Savitz was also special in that both unipolar and bipolar depressed patients were included.

**Table 1C** additionally shows that there are also two out of three studies (57, 58) that showed that in the case of add-on *n*-acetyl cysteine, it was of no use to stratify the patients in low- or high-grade inflammation prior to therapy. Two of the studies of add-on *n*-acetyl cysteine (one showing and one not showing an effect of prior determination of the inflammatory state) involved both unipolar and bipolar depressed patients.

It was remarkable that when an add-on anti-inflammatory agent was given to patients with low signs of inflammation, reduced responses were obtained as compared to patients with high signs of inflammation and even to placebo (two out of three of such studies) (53, 54). Also, when fish oil (an agent with both lipid-correcting and anti-inflammatory properties) was given as a monotherapy to patients with a low inflammatory state, reduced responses were seen as compared to placebo ( (**Table 1C**). As mentioned above, add-on aspirin did induce an increased response in patients with low signs of inflammation in the study of Savitz et al. (54).

Taking these literature data together, it is difficult to draw a simple conclusion on the usefulness of a prior measurement of serum inflammatory markers for the determination of the effect of (add-on) anti-inflammatory agents. There is a clear trend that (add-on) anti-inflammatory agents, such as infliximab, minocycline, and fish oil are effective if inflammatory markers are clearly present, but this does not apply to aspirin and *n*-acetylcysteine. However, it is also safe to say that special caution must be given when there is an absence of circulating inflammatory markers in patients with MDD: the chances are high that the use of the effective anti-inflammatory agents (such as infliximab, minocycline, and fish oil) in states of moderate–high inflammation actually has an opposite effect than expected in such patients, namely, a reduced responsiveness.

### Systematic Review Data on Gene Expression in Circulating Leukocytes

**Table 2** shows the studies we selected that dealt with the gene message for pro-inflammatory cytokine production in the circulating leukocyte pool prior to treatment and predictive for treatment outcome. We found five relevant articles.

In 2013, Powell et al. (59) described a significantly increased baseline expression of *TNF* in escitalopram non-responders (*n* = 21) compared to responders (*n* = 25) taken from the GENDEP study. In the same year, Cattaneo et al. (61) reported on data of the GENDEP study and found higher baseline mRNA levels for *IL1β*, macrophage inhibiting factor (*MIF*), and *TNF* in antidepressant (escitalopram or nortriptyline) non-responders compared to responders, the three cytokine expressions together explaining 46% of the variance of treatment response.

Belzeaux et al. (63) identified an algorithm of four mRNAs, including two cytokine genes (TNF and *IL1β*, together with *PPT1* and *HIST1H1E*) to be predictive of the treatment response in MDD. However, the weakness of their study was that a whole scale of antidepressants was used, while numbers of patients and HC were limited (16 vs. 13). Guilloux et al. (60) predicted non-remission following escitalopram treatment in MDD with an accuracy of 79.4% using a 13-gene model including four genes associated with immune and inflammatory activation (however, *TNF* was not part of the 13 genes). Mediation of cell proliferation was another important function of the remaining genes, but not exclusively. In 2016, Cattaneo et al. (62) took the data of the GENDEP study further and reported that absolute values of the message for *IL1β* and *MIF* together could predict non-responsiveness to escitalopram or nortriptyline in over 99%. These outcomes were confirmed in an independent, naturalistic replication sample.

Taken together, it is clear that non-responsiveness to an SSRI or to a TCA (nortriptyline) can likely be predicted by determining the expression level of combinations of important immune genes (*IL1β*, *MIF*, *TNF*, and *CD3*) in preparations of circulating leukocytes of patients with MDD.

### Experimental Data on Inflammation-Related Gene Expression in Circulating Monocytes as a Predictor of Treatment Response

Prior to treatment, we could test 34 patients with MDD [mean age: 52.2 (±9.9) years, 59% females, collected at the ErasmusMC, Rotterdam] for inflammatory gene expression in their circulating monocytes. As a control group, we tested 45 HC of comparable age [mean age: 49.1 (±9.4) years] and gender (44% females). Of the 34 patients, 14 were treated with venlafaxine and 20 were treated with imipramine. An overall response rate of 11% was found in this trial, with 11/34 patients responding to treatment. The difference between the response rates for both treatment arms were not statistically significant, i.e., a response rate of 36% (5/14) for patients treated with venlafaxine and of 30% (6/20) for patients treated with imipramine. Vermeiden et al. (38) have reported extensively on this study and described that in the entire group of patients (*n* = 85), 45% of the patients responded to this first line of drug treatment (measured as 50% HAM-D reduction).

The other series of patients involved 35 patients with MDD [mean age: 41.4 (±10.8) years, 46.7% females, collected at the LMU, Munich] and 42 HC of comparable age [mean age: 37.9 (±11.9) years] and gender (61.9% females). Of the 35 patients, 19 were treated with sertraline *plus* placebo and 16 were treated with sertraline *plus* celecoxib. A high overall response rate was found in this trial, i.e., 26/35 (74.3%) of patients responded to treatment. The difference between the response rates for both treatment arms was not statistically significant, i.e., a response rate of 68.4% (13/19) for patients treated with sertraline *plus* placebo and 81.3% (13/16) for patients treated with sertraline *plus* celecoxib.

We determined with an already published algorithm [see the Section Monocyte Inflammatory Gene Expression and Ref. (45)] the inflammatory state of the monocytes using the top 10 cluster 1 inflammatory genes (*IL1β*, *CCL20*, *EREG*, *IL6*, *TNFAIP3*, *CXCL2*, *PDE4B*, *ATF3*, *PTX3*, and *IL1A*). We controlled the patient monocyte tests with the outcomes of the same tests carried out in HC. **Table 3** shows that, in each study group, a significant larger proportion of patients had—prior to therapy—circulating monocytes with a positive inflammatory gene signature as compared to the respective HC. Taking all patients from the four study groups together, 25 of the 69 (36%) patients with MDD had circulating monocytes with a pro-inflammatory gene signature, while only 9 of 87 (10%) HC had such monocyte signature (*p* < 0.05). This observation is in accord with earlier observations that monocytes of part of the patients with MDD show signs of a high inflammatory state (21).

For the purpose of this study, we divided the total patient group in those with a negative monocyte inflammatory gene score and those with a positive score. The data in **Table 3** show that in the response rates in three out of four patient groups, patients with a positive inflammatory gene score had a lower response rate than those without a positive score. This, however, did not apply to the sertraline *plus* placebo group, and also significant differences were not reached in any of the groups. The phenomenon of better responsiveness in “non-inflamed” MDD patients could also be seen in the total MDD patient group; patients with a positive inflammatory gene score had a lower response rate than patients without a positive inflammatory gene score (i.e., 44% vs. 59%); however, a statistical significance was not reached in the total group of patients.

## Discussion

### The Predictive Capability of the Inflammatory State for Anti-Depressive and Anti-Inflammatory Treatment and Potential Mechanisms

The data of the systematic review and experimental monocyte data, as presented in this study, collectively point in the direction that the state of so-called low-grade inflammation does play a role in the outcome of antidepressant therapy of patients with MDD.

Low-grade inflammation is characterized by an increase in the serum level of pro-inflammatory compounds (e.g., CRP, IL-1 β, IL-6, and TNF-α) and/or an activation state of circulating or tissue resident immune cells, including the brain microglia. Both an increase in pro-inflammatory compounds in the blood of patients with MDD and a pro-inflammatory activation of microglia and/or of myeloid cells in the periphery (i.e., monocytes) have been documented in a considerable (≈30–40%) proportion of patients with MDD (20, 73, 74). Moreover, imaging and histological techniques have shown microglial activation in the hippocampus of depressed patients (75).

By producing an array of neurotrophic factors, pro- and anti-inflammatory cytokines (e.g., IL-6), as well as axon guidance molecules, non-inflammatory activated microglia has been implicated both in white matter integrity and in the adequate development and function of important stress-regulating systems in the healthy brain (76, 77). On the contrary, inflammatory activated microglia (and/or a transfer of peripheral pro-inflammatory cytokines to the brain) is thought to hamper the normal development, growth, and synaptic function of stress-regulating systems and brain connections important for mood regulation, such as the white matter tracts between the forebrain and the limbic system. To illustrate this, raised serum pro-inflammatory cytokine levels have been associated in mood disorder patients with increased activation of threat- and anxiety-related neuro-circuits (78), reduced neural responses to negative stimuli in frontal brain regions involved in cognitive and emotional functions (79), and compromised integrity of myelin sheaths in cortico-limbic networks involved in mood regulation (80).

Importantly, low-grade inflammation has been shown to influence not only brain development and function but also neurotransmission, with excellent reviews on the inhibitory effects of pro-inflammatory cytokines, such as IL-1β and TNF-α, on the synaptic availability of monoamines and BDNF, while the same cytokines have been shown to increase extracellular glutamate, all important molecular determinants in MDD pathogenesis and response to treatment (15).

The data from the here presented systematic review on circulating inflammatory compounds indicate that patients with MDD with an activated inflammatory state (as measured by, e.g., moderate to high levels of circulating CRP, IL-6, and/or TNF-α) show reduced response rates to antidepressant regimens with a primarily serotonergic action (e.g., escitalopram), while showing improved response rates to antidepressant regimens with a primarily noradrenergic (e.g., nortriptyline), dopaminergic (e.g., bupropion, mirtazapine), or glutamatergic action (i.e., ketamine).

The systematic review data on the inflammatory gene expression in circulating leukocytes confirmed this phenomenon, showing that patients with a high gene expression level of *IL1β*, *TNF*, and/or *MIF* did not respond well to interventions with an SSRI in comparison to MDD patients with a low expression of these genes. However, gene data in circulating leukocytes disagreed with the data reported for circulating inflammatory compounds regarding TCA. While the primarily noradrenergic TCA nortriptyline did not give a satisfactory response in patients with MDD with a high gene expression level in circulating leukocytes (see **Table 2**), nortriptyline did in patients with high levels of circulating inflammatory compounds (see **Table 1B**).

Apparently, inflammatory gene expression in leukocytes does not measure the same level of inflammation than the measurement of circulating inflammatory compounds in serum/plasma; a high inflammatory gene expression might typify a state of “stronger/other” inflammation in MDD needing a treatment with drugs beyond the serotonergic and noradrenergic drugs. In other studies, we have also noted that inflammatory gene expression in circulating cells does not correlate one to one with the circulating protein gene product in serum/plasma (81). We explained this phenomenon by assuming that resident cells, such as the endothelial cells and resident macrophages in the tissues, also contribute to the level of circulating inflammatory compounds.

Although the data of our experiments on inflammatory gene expression levels in circulating monocytes (a subset of the circulating leukocytes) did not deliver statistically significant results, they were, by and large, in agreement with the above-described findings for the gene expression in all circulating leukocytes and showed that patients with “inflammatory” monocytes showed reduced response rates to predominantly serotonergic drug interventions and patients with “non-inflammatory” monocytes showed higher response rates to these type of agents.

Collectively, we deduce from these data that MDD patients with an activated inflammatory state (as measured by moderate to high circulating levels of, e.g., CRP, TNF-α, and IL-6, or a high gene expression of, e.g., *IL1β*, *TNF*, and *MIF* in circulating leukocytes) need more than a monotherapy with a predominantly serotonergic agent to improve clinically in a satisfactory way. An option then seems to be an immediate step up to agents with also a strong dopaminergic or glutamatergic action.

The reason for a better response to dopaminergic or glutamatergic drugs in the case of signs of enhanced inflammation can only be speculated on. It is possible that these drugs are needed because they also have clear anti-inflammatory actions, counteracting the detrimental effects of the high inflammatory state on the signs and symptoms of depression. There is ample evidence that dopamine and ketamine can reduce the production of pro-inflammatory cytokines and enhance that of anti-inflammatory cytokines (82, 83). On the other hand, the pro-inflammatory state itself may lead to an altered neurotransmitter metabolism, necessitating more than a primarily serotonin reuptake inhibition, but also an intervention in the dopamine or glutamate metabolism. Pro-inflammatory cytokines have been reported to activate neuronal mitogen-activated protein kinase (MAPK) pathways, increasing monoamine transporter expression and activity in general, which leads to an increased pre-synaptic reuptake of not only serotonin but also other neuroactive amines (84, 85). Furthermore, the state of enhanced inflammation is thought to lead to an enhanced tryptophan breakdown *via* the kynurenine pathway, resulting in various neuroactive compounds, among which NMDA agonists and antagonists, aggravating glutamatergic neurotransmitter imbalances (86, 87). This might also necessitate more than only a serotonin reuptake inhibition to be effective.

A step up to dopaminergic and glutamatergic antidepressants was more effective in “inflammatory” MDD patients than in “non-inflammatory” patients, and a combination of an antidepressant with an anti-inflammatory agent increased the response rates in these “inflammatory” patients with MDD as compared to “non-inflammatory” MDD patients. Though the reviewed literature data are scarce, the best prediction results seem to be obtained for infliximab (anti-TNF-α agent), minocycline (tetracycline), and eicosapentaenoic acid (fish oil). For *n*-acetylcysteine, the inflammatory state did show conjectural prediction effects, while for aspirin (acetylsalicylic acid), a reduced response was actually seen in “inflammatory” patients as compared to “non-inflammatory” patients.

The strength or character of the anti-inflammatory agents may have played a role in this variation of predictability of the state of inflammation for the (add-on) anti-inflammatory agents. Both anti-TNF agents and minocycline are in clinical practice and are considered stronger anti-inflammatory drugs than *n*-acetylcysteine and aspirin. However, for fish oil, a high inflammatory state was also predictive for a better effect in MDD patients, while fish oil is considered a relatively weak anti-inflammatory agent. Interestingly, fish oil exerts its anti-inflammatory effects *via* changing the “bad” pro-inflammatory lipid state of individuals (88), and perhaps the high state of inflammation in MDD patients is primarily driven by a bad lipid profile, which is then best corrected by fish oil.

Also, direct or indirect neurotransmitter effects of the anti-inflammatory agents may have played a role in the success or failure to predict their improved responsiveness in “inflammatory” MDD patients. Interestingly, two of the three add-on anti-inflammatory agents (minocycline and fish oil) that worked better in “inflammatory” than in “non-inflammatory” patients possess dopaminergic activities (89, 90). *N*-acetylcysteine, of which it is conjectural whether it works better as add-on in “inflammatory” than in “non-inflammatory” MDD patients, influences both dopamine and glutamate levels in the brain (91). Add-on aspirin, in contrast, had fewer effects in “inflammatory” MDD patients as compared to “non-inflammatory” patients; interestingly, aspirin has anti-glutamatergic actions (92, 93). These varying neuro-modulating actions of anti-inflammatory drugs make complex interactions in the neuro-immune network possible, inducing varying outcomes of combinations of antidepressants and anti-inflammatory agents. Of note also is that three of the reviewed studies of add-on anti-inflammatory agents had included bipolar depressed patients (54, 56, 58). This applies in particular to the study on aspirin (54), in which a reducing effect was found in “inflammatory” versus “non-inflammatory” patients. Intrinsic differences between bipolar and unipolar depression, such as differences in the immune and the glutamate state (94–98), may have played a role here.

Despite the above-listed uncertainties, it is nevertheless tempting to postulate—based on the outcomes of the literature review—that when MDD patients are “inflammatory”, (add-on) anti-inflammatory drugs are also an option to improve responsiveness and then the best results are probably obtained when anti-inflammatory agents are potent, influence lipid metabolism, and/or influence primarily dopaminergic synaptic transmission.

Regarding the use of (add-on) anti-inflammatory agents, another important message emerges from our systematic review of the literature. Interestingly, three out of four reports (52–54) indicated that “non-inflammatory” MDD patients showed a reduced response rate as compared to even placebo to the effective (add-on) intervention with an anti-inflammatory agent. In other words, the addition of the anti-inflammatory drugs effective in “inflammatory” patients was detrimental, and the anti-inflammatory drugs inhibited the effect of the antidepressants or delayed natural recovery. Such an outcome of an anti-inflammatory regimen is counterintuitive, if one assumes that inflammation contributes to depressive symptomatology (see before). The authors of one of the papers describing this phenomenon (53) explain their finding, that perhaps a small activation of the inflammatory system is needed for mental well-being and that both an extreme low and an extreme high activity of the inflammatory response system is disadvantageous for mental health. In other words, there would be an optimal set point for the inflammatory state of an individual for mental health. Downregulating this optimal state with an effective anti-inflammatory agent would, in such a view, be counterproductive and would open the way for the development of depressive symptoms.

Another explanation is that there exists a form of MDD that is non-immune and characterized by absent serological markers of immune activation. As indicated, (add-on) aspirin has a beneficial effect in patients and it can be hypothesized that it is in particular the neuro-modulating effect (anti-glutamatergic) of aspirin that induces this effect.

Based on this literature review, what appears to be the best and easiest assay system to measure the inflammatory state of MDD patients? The systematic review data on the gene expression level of cytokines in circulating leukocytes showed that two of the leukocyte gene expression studies resulted in very good accuracy rates of prediction of non-responsiveness, and algorithms could be developed, which showed high accuracies from 75% to even a 100% to predict non-responsiveness to an SSRI/TCA drug intervention (60, 62). Apparently, high levels of inflammatory cytokine gene message in circulating leukocytes are a precise sign of poor (treatment) outcome, and perhaps even better than high levels of inflammatory compounds/cytokines in serum/plasma. Nevertheless, it is technically less demanding to measure inflammatory compounds/cytokines in serum/plasma than to perform a gene expression assay in circulating leukocytes. Regarding the inflammatory markers best to be measured in serum/plasma to determine a raised inflammatory state in patients with MDD, it is worthy to note that the most consistent effects were found in our literature analysis with circulating CRP and/or IL-6 levels. These inflammatory compounds were tested in a large proportion of the here reviewed studies on serum inflammatory compounds (15/19), and outcomes and conclusions were congruent between these studies regarding these two inflammation markers.

Circulating TNF-α was measured in only six studies; hence, sufficient information on the validity of this parameter is lacking. Importantly, one of the studies showed that circulating TNF-α levels were not in agreement with the general rule, finding that a high TNF-α level was not predictive of a decreased responsiveness to an SSRI/SNRI (while a high IL-6 level in the same study was) (43). Other circulating inflammatory compounds (e.g., IL-8, IL-10, and IL-1) have also been tested in the here reported studies, but in only very few studies, and therefore data cannot be reliably evaluated. They nevertheless showed the general trend for serum/plasma factors that, in a state of inflammation, more than a monotherapy with a predominantly serotonergic agent might be needed.

Collectively, it seems that for predicting responsiveness to regular antidepressants, the avenue exploring the usefulness of serum/plasma CRP and IL-6 determination is the easiest and clinically the most feasible and promising approach. High levels of CRP/IL-6 would indicate that treatment with a serotonergic drug is not effective enough. However, the data reviewed here also indicate that the gene expression in circulating leukocytes cannot be neglected as a predicting parameter due to the reported high levels of accuracy to predict non-responsiveness to SSRI/SNRI and TCA therapy.

### Limitations

In this article, we only focused on inflammation parameters as determinants for the outcome of treatment. The various other determinants important for treatment outcome have recently been reviewed by Perlman et al. (99). The authors described not only that inflammation-related determinants are important but also that a whole array of genetic, endocrine, neuroimaging, sociodemographic, and symptom-based predictors turn out to influence outcome. However, due to heterogeneous sample sizes, effect sizes, publication biases, and methodological disparities across reviews, Perlman et al. (99) concluded that they could not accurately assess the strength and directionality of the predictors, and the authors therefore highlighted the importance of large-scale research initiatives and the use of clinically easily accessible biomarkers, as well as the need for replication studies of current findings. Clearly, we support such view and underscore the notion that our review data are also affected by the heterogeneous sample sizes, effect sizes, publication biases, and methodological disparities and that the data do not yet give a clear-cut picture. Also, our own experimental data on monocyte gene expression were underpowered and too limited to obtain clear-cut results and significances. Thus, clearly more studies are needed using standardized add-on anti-inflammatory treatments to standardized single antidepressant medications to develop a clearer picture of the actual response rates in immune and otherwise stratified patients with MDD.

### Conclusions

There are excellent recent reviews on the discovered signs of low-grade inflammation in psychiatric patients that have transformed our understanding of neuropsychiatric diseases and urge for new diagnostic and therapeutic criteria in the emerging field of immuno-psychiatry (100). There are, however, at present, insufficient data and reliable concepts on the inflammation pathogenesis of MDD to design clinically applicable treatment drug protocols with reliable cutoff points for inflammatory parameters to guide therapy regimens.

Despite this limitation, a few generalizations can nevertheless be made from our study regarding inflammation as a predictor. Of the inflammation parameters, the serum CRP and IL-6 seem to be the most promising parameters for further clinical development. They are relatively easy to determine and, thus, useful in clinical studies. Using these parameters, a state of raised inflammation (as evidenced by raised serum CRP and IL-6 levels) characterizes a form of MDD with a relatively poor outcome and a non-responsiveness to agents with a predominant serotonergic action. Such cases might need a faster step-up to drug regimens with agents with dopaminergic (e.g., mirtazapine and bupropion) or glutamatergic (e.g., ketamine) effects, or a combination of a first-line antidepressant with an anti-inflammatory agent such as infliximab, minocycline, or fish oil (but not aspirin), most of them showing dopaminergic action. Varying anti-inflammatory properties of antidepressants as well as varying neuro-modulatory effects of anti-inflammatory agents (and/or complex interactions thereof) may play a role in the therapeutic success or failure of the step-ups.

A word of caution is needed regarding the regimens using as add-on the successful anti-inflammatory agents infliximab, minocycline, and fish oil: There must indeed be laboratory signs of inflammation (i.e., raised serum levels of CRP or IL-6) for this addition to be effective. If not, even response rates lower than the non-add-on situation might be obtained.

## Ethics Statement

These studies were carried in compliance with standards of CGP, assuring that the rights, safety, and well-being of patients were protected in accordance with the principles that have their origin in the Declaration of Helsinki (June 1964, last amendment Fortaleza 2013). Additionally, the relevant national and European regulations were adhered, too. After study procedures had been fully explained, all subjects provided written informed consent.

## Author Contributions

GA and MS designed the strategy of the present review. BB and EW collected part of the study cohort, GA, AW and HD evaluated the data. GAH and MS wrote the first draft of the paper, HD and NM contributed with supervision and expert advice and critically revised the draft. All other authors contributed to the manuscript revision and approved the submitted version.

## Funding

This study was financially supported by the EU *via* the MOODINFLAME project (EU-FP7-HEALTH-F2-2008-222963), the PSYCHAID (EU-FP7-PEOPLE-2009-IAPP-MarieCurie-286334), and the MOODSTRATIFICATION project (H2020-EU.3.1.1., GA754740). NM and GA were additionally supported by the foundation “Immunität und Seele.” The funders had no role in study design, data collection, analysis and interpretation of data, the writing of the report, and the decision to submit the paper for publication.

## Conflict of Interest Statement

The authors declare that the research was conducted in the absence of any commercial or financial relationships that could be construed as a potential conflict of interest.
